# Transcriptional and post-translational regulation of plant autophagy

**DOI:** 10.1093/jxb/erad211

**Published:** 2023-06-26

**Authors:** William Agbemafle, Min May Wong, Diane C Bassham

**Affiliations:** Roy J. Carver Department of Biochemistry, Biophysics and Molecular Biology, Iowa State University, Ames, IA, USA; Department of Genetics, Development and Cell Biology, Iowa State University, Ames, IA, USA; Department of Genetics, Development and Cell Biology, Iowa State University, Ames, IA, USA; Universidad de Sevilla, Spain

**Keywords:** ATG, autophagy, gene expression, persulfidation, phosphorylation, post-translational modification, starvation, stress, transcription factors, ubiquitination

## Abstract

In response to changing environmental conditions, plants activate cellular responses to enable them to adapt. One such response is autophagy, in which cellular components, for example proteins and organelles, are delivered to the vacuole for degradation. Autophagy is activated by a wide range of conditions, and the regulatory pathways controlling this activation are now being elucidated. However, key aspects of how these factors may function together to properly modulate autophagy in response to specific internal or external signals are yet to be discovered. In this review we discuss mechanisms for regulation of autophagy in response to environmental stress and disruptions in cell homeostasis. These pathways include post-translational modification of proteins required for autophagy activation and progression, control of protein stability of the autophagy machinery, and transcriptional regulation, resulting in changes in transcription of genes involved in autophagy. In particular, we highlight potential connections between the roles of key regulators and explore gaps in research, the filling of which can further our understanding of the autophagy regulatory network in plants.

## Teaser

Autophagy is tightly controlled by environmental and developmental cues. This review highlights key regulatory mechanisms that modulate autophagy through post-translational modification or transcriptional regulation.

## Introduction

Plant macroautophagy (hereafter termed autophagy) is a catabolic pathway that degrades and recycles unwanted cellular components such as aggregated proteins and dysfunctional organelles (Marshall and Vierstra, 2018). Under optimal growth conditions, autophagy occurs at a low basal level and operates as a housekeeping mechanism to maintain cellular homeostasis and promote plant development. Under stressful conditions, where plant survival is threatened, autophagy is up-regulated to relieve the burden imposed by the stress. This stress-induced autophagy results in the bulk degradation and recycling of different types of cytoplasmic cargo (Signorelli *et al.*, 2019). Autophagy can also be selective by targeting specific cellular components. For example, autophagy facilitates the quality control of specific organelles including mitochondria (Nakamura *et al.*, 2021), chloroplasts (Izumi *et al.*, 2017) and endoplasmic reticulum (Liu *et al.*, 2012). Autophagy is induced by different types of stresses (Signorelli *et al.*, 2019) and is also a key mechanism for nutrient remobilization (Thompson *et al.*, 2005; Wada *et al.*, 2009, 2015; Guiboileau *et al.*, 2012, 2013) and pathogen defense (Liu *et al.*, 2005; Patel and Dinesh-Kumar, 2008; Lenz *et al.*, 2011; Kwon *et al.*, 2013), emphasizing its importance in plant metabolism, immunity, and stress response.

**Fig. 1.**
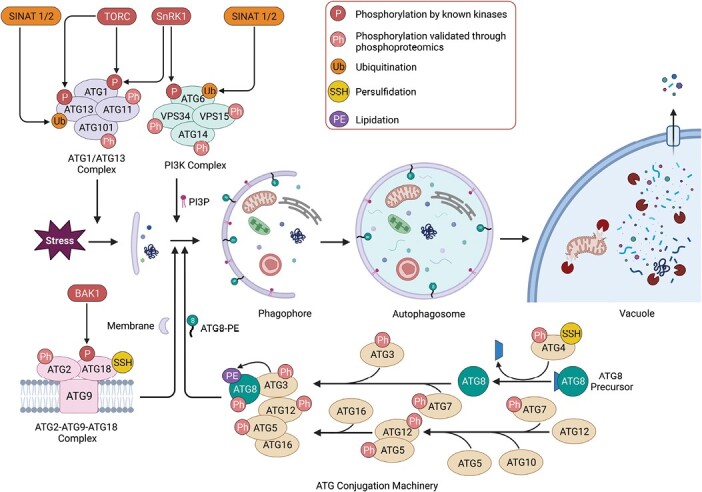
Post-translational modifications (PTMs) of core ATG proteins in plants. During autophagy, the core ATG proteins form distinct functional groups that can be divided into the ATG1/ATG13 kinase complex, PI3K complex, ATG2–ATG18–ATG9 complex, and the ATG conjugation machinery. Post-translational modifications such as phosphorylation, persulfidation, ubiquitination, and lipidation occur in the early steps of autophagy and dictate the function, dynamics, and stability of these proteins. Stress triggers the ATG1/ATG13 kinase complex to initiate phagophore formation. The PI3K complex decorates the phagophore with PI3P to facilitate vesicle nucleation while the ATG2–ATG9–ATG18 complex provides lipids and membranes to promote phagophore expansion. The ATG8 and ATG12 conjugation systems together facilitate ATG8 lipidation to promote autophagosome maturation. The completed autophagosome fuses with the vacuole to deposit the cargo for degradation and recycling. ‘P’ refers to phosphorylation known to be regulated by the indicated upstream kinase and ‘Ph’ refers to phosphorylation validated through phosphoproteomics by Mergner *et al.* (2020) and Montes *et al.* (2022).

Autophagy involves a series of molecular events mediated by a group of highly conserved genes termed *AuTophaGy-related* (*ATG*) genes, which in plants often occur as gene families. The process begins with the formation of a phagophore, a cup-shaped double-membrane structure that forms around the cellular cargo. The phagophore matures to enclose and isolate the entire cargo, resulting in the formation of a double-membrane vesicle called an autophagosome (Wun *et al.*, 2020). Autophagosome biogenesis is facilitated by four functional groups of proteins: (i) the ATG1/ATG13 kinase complex initiates phagophore formation (Suttangkakul *et al.*, 2011; Li *et al*., 2014), (ii) the phosphatidylinositol 3-kinase (PI3K) complex facilitates vesicle nucleation by decorating the growing phagophore with phosphatidylinositol-3-phosphate (PI3P) (Liu *et al*., 2020; Bhati *et al.*, 2021), (iii) an ATG2–ATG9–ATG18 complex may promote phagophore membrane expansion and modulate autophagosome progression from the endoplasmic reticulum (Xiong *et al.*, 2005; Zhuang *et al.*, 2017; Kang *et al.*, 2018), and (iv) the ATG8 and ATG12 ubiquitin-like conjugation systems mediate phagophore expansion and maturation (Marshall and Vierstra, 2018). Once complete, the mature autophagosome fuses with a nearby lytic vacuole, depositing the cargo into the vacuolar lumen to be degraded by hydrolases. The resulting products of this digestion are transported from the vacuole into the cytoplasm to be reused (Soto-Burgos *et al.*, 2018) (Fig. 1).

Within the last decade, significant progress has been made in characterizing the functional and structural roles of the various components of the plant autophagy process and in deciphering the diverse roles autophagy plays in plant health, development and responses to biotic and abiotic stresses. In addition, research focusing on understanding the mechanisms that facilitate autophagy regulation has recently garnered attention with a number of regulatory factors being identified to function in response to specific conditions and signals. However, the mechanisms by which these different factors are coordinated to appropriately integrate specific signals with autophagy regulation and plant resilience to stress is largely unexplored. This review aims to summarize recent advances in understanding key regulatory factors that modulate plant autophagy at the transcriptional and post-translational levels and further highlights the importance of their roles in the autophagy regulatory network.

## Post-translational regulation of plant autophagy

Post-translational modification is a process in which the amino acids of target proteins are covalently modified with chemical groups, for example phosphate, ubiquitin, persulfide, acetate or methyl, leading to changes in the characteristics of the proteins. The process of autophagosome biogenesis is tightly modulated by the core ATG proteins and the function, dynamics, and stability of these proteins are regulated through post-translational modifications (Fig. 1). Here, we discuss the various roles that such modifications play in plant autophagy regulation.

### Regulation of autophagy by phosphorylation

Protein phosphorylation is the most extensively studied post-translational modification that regulates autophagy (H. Li *et al*., 2022b; Licheva *et al.*, 2022). Modulation of protein phosphorylation can influence protein activity, interactions, subcellular localization, and stability (Seet *et al.*, 2006). Most phosphorylation events in plants occur on serine and threonine residues, with a much smaller portion on tyrosine (Champion *et al.*, 2004). Occasionally, histidine and arginine can also be phosphorylated (Durek *et al.*, 2010; van Wijk *et al.*, 2014). As in other organisms, the conserved kinases TARGET OF RAPAMYCIN COMPLEX (TORC) and SUCROSE NON-FERMENTING-RELATED KINASE 1 (SnRK1), an ortholog of ADENOSINE MONOPHOSPHATE (AMP)-ACTIVATED PROTEIN KINASE (AMPK) in mammals and SUCROSE NON-FERMENTING 1 (Snf1) in yeast, play a critical role in controlling plant autophagy (Liu and Bassham, 2010; Chen *et al*., 2017; Pu *et al.*, 2017a, b; Soto-Burgos and Bassham, 2017). In addition, BRASSINOSTEROID INSENSITIVE 1-ASSOCIATED RECEPTOR KINASE 1 (BAK1) (Zhang *et al.*, 2021) and BRASSINOSTEROID-INSENSITIVE 2 (BIN2), which are involved in brassinosteroid (BR) signaling, can phosphorylate autophagy-related substrates to regulate autophagy under stress (Nolan *et al.*, 2017; Zhang *et al.*, 2021; Liao *et al.*, 2023; Montes *et al.*, 2022). While dephosphorylation by protein phosphatases is also expected to be important in the regulation of autophagy, this is relatively less studied in plants (Ahn *et al.*, 2011; Wang *et al*., 2022).

#### TORC is a negative regulator of autophagy

TORC is a central regulator in energy and nutrient sensing during plant growth and development (Rodriguez *et al.*, 2019; Li *et al*., 2021). Arabidopsis TORC is composed of the TOR kinase catalytic subunit, REGULATORY-ASSOCIATED PROTEIN OF TOR (RAPTOR) and LETHAL WITH SEC13 PROTEIN 8 (LST8). RAPTOR recruits substrates for TOR to phosphorylate whereas LST8 promotes the stability of the TOR complex (Mugume *et al.*, 2020).

**Fig. 2.**
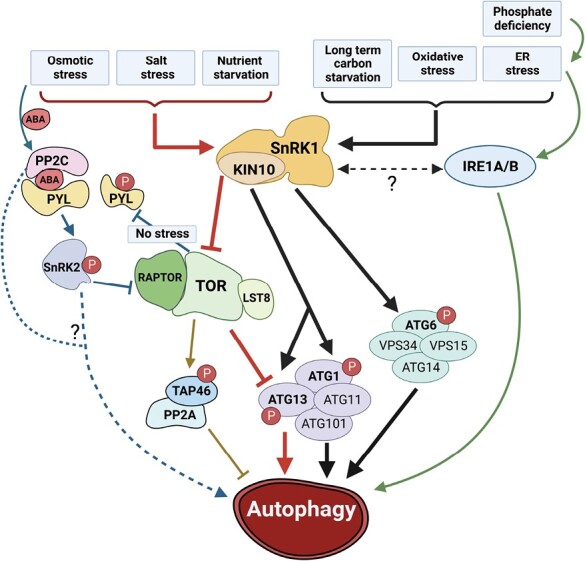
Regulatory pathways that modulate plant autophagy in response to stress conditions. Autophagy is induced by different stress conditions via TORC-dependent (red arrow) and TORC-independent pathways (black arrow). Nutrient starvation, salt, and osmotic stress activate SnRK1, which inhibits TORC expression and activity. TORC can suppress autophagy through the inhibition of the ATG1/ATG13 complex (red arrow). SnRK1 can directly phosphorylate and activate the ATG1/ATG13 complex, leading to autophagy induction. Upon long-term carbon starvation, SnRK1 phosphorylates ATG6 to activate autophagy, which is independent of TORC (black arrow). Endoplasmic reticulum stress and oxidative stress activate autophagy through SnRK1-mediated phosphorylation of ATG1, independent of TORC repression (black arrow). Endoplasmic reticulum stress-induced autophagy is also regulated by INOSITOL REQUIRING 1A/B (IRE1A/B), but its relationship with SnRK1 is unknown (black dashed arrow). Phosphate deficiency activates autophagy through the endoplasmic reticulum stress-mediated pathway (green arrow). TAP46 (a regulatory subunit of PP2A) is phosphorylated by TORC, acts as a downstream effector of TOR signaling, and negatively regulates autophagy (beige arrow). All the stress-induced autophagy pathways require SnRK1 activity. Upon osmotic stress, ABA-activated SnRK2 phosphorylates RAPTOR and inhibits TORC activity. In the absence of stress, TORC phosphorylates the PYL ABA receptors (blue arrow). Whether or not SnRK2 kinase and PP2C protein phosphatase are involved in autophagy regulation through TORC inhibition is still unknown (blue dashed arrow).

TORC is a negative regulator of autophagy in yeast and animals (Noda and Ohsumi, 1998; González and Hall, 2017), and this role is conserved in plants (Liu and Bassham, 2010; Soto-Burgos and Bassham, 2017). Arabidopsis *RNAi*-TOR knockdown lines and a *raptor1b* mutant exhibit constitutively active autophagy and have increased expression of some *ATG* genes (Liu and Bassham, 2010; Pu *et al.*, 2017a; Soto-Burgos and Bassham, 2017). Conversely, both TOR overexpression and auxin-mediated activation of TOR repress autophagy under certain stress conditions but not others (Pu *et al.*, 2017a, b). Hence, the repression of autophagy by TOR is dependent on the type of upstream stress. TOR represses autophagy under normal conditions, but in some stresses, such as nutrient deprivation, salt stress, and osmotic stress, this repression must be relieved for autophagy to be activated. In contrast, upon oxidative stress or endoplasmic reticulum stress, autophagy can be activated even in the presence of high TOR activity (Mahfouz *et al.*, 2006; Pu *et al.*, 2017a). It is unclear why TOR inactivation leads to autophagy activation in certain stresses, whereas in other stresses, autophagy is activated regardless of TOR activity. It is appealing to speculate that different stresses may give rise to distinct pathways that might activate autophagy via TOR-dependent and TOR-independent mechanisms (Fig. 2).

In mammals, TOR blocks the activation of the ATG1 homolog UNC51-LIKE AUTOPHAGY ACTIVATING KINASE 1 (ULK1) under nutrient-rich conditions through the phosphorylation of the ULK1 Ser757 residue (Kim *et al.*, 2011) and in yeast, TOR hyperphosphorylates ATG13 to inhibit autophagy (Kamada *et al.*, 2010). Quantitative phosphoproteomics show that ATG1 and ATG13 are phosphotargets of the Arabidopsis TORC (Van Leene *et al.*, 2019; Montes *et al.*, 2022). Under nutrient-rich conditions, RAPTOR recruits ATG13 through the plant TOR-signaling (TOS) motif (Son *et al.*, 2018). This allows TOR to directly phosphorylate ATG13, possibly reducing ATG1 activity, and leading to the suppression of autophagy (Suttangkakul *et al.*, 2011; Van Leene *et al.*, 2019). The phosphorylation of ATG13 is reduced when the TOS motif is disrupted (Son *et al.*, 2018), confirming the importance of TOR in ATG13 regulation. ATG13 is thus directly phosphorylated by TOR to inhibit autophagy in plants.

While a large number of upstream regulators of mammalian TORC are known (González and Hall, 2017), the majority cannot be identified in plant genomes, suggesting distinct mechanisms for regulation of plant TORC activity. Only a few factors have been shown to regulate TORC in plants (see below); the identification of additional upstream regulators of TORC therefore remains a critical area for future research.

#### SnRK1 is a positive regulator of autophagy

Arabidopsis SnRK1 is a heterotrimeric complex consisting of a protein kinase/catalytic (α) subunit and two regulatory (β and γ) subunits (Crozet *et al.*, 2014). The catalytic subunit of Arabidopsis SnRK1 is encoded by *KIN10*, *KIN11* and *KIN12*. KIN12 appears to be non-functional and poorly expressed while KIN10 and KIN11 are partially redundant and well-expressed (Baena-González *et al.*, 2007). The majority of SnRK1 function is associated with KIN10 catalytic activity (Jossier *et al.*, 2009).

SnRK1 and TORC function antagonistically in the regulation of nutrient responses (Robaglia *et al.*, 2012; Baena-González and Hanson, 2017). In yeast and mammals, autophagy is negatively regulated by TORC and positively regulated by SnRK1 homologs (Kim *et al.*, 2011; González *et al.*, 2020), and this inverse regulation is conserved in plants (Pu *et al.*, 2017a, b; Soto-Burgos and Bassham, 2017). SnRK1 is required for autophagy in all the stress conditions indicated in Fig. 2, whether dependent upon TORC repression or not. Overexpression of KIN10 leads to constitutive activation of autophagy whereas autophagy is blocked in a *kin10* mutant (Chen *et al*., 2017; Soto-Burgos and Bassham, 2017). KIN10 inhibits TORC activity to induce autophagy (Soto-Burgos and Bassham, 2017). KIN10 interacts with and directly phosphorylates RAPTOR1B *in vitro* (Nukarinen *et al.*, 2016), suggesting that KIN10-mediated phosphorylation of RAPTOR1B inactivates TORC to promote autophagy, possibly by blocking the ability of RAPTOR1B to recruit autophagy-related substrates. Inhibiting TORC activity significantly activates autophagy in the *kin10* mutant, in which otherwise autophagy is blocked under both normal and stress conditions. In addition, increasing TORC activity suppresses KIN10-induced autophagy activation (Soto-Burgos and Bassham, 2017). Together, these data suggest that TORC acts downstream of SnRK1 to regulate autophagy in conditions in which autophagy is dependent on TORC repression.

AMPK promotes autophagy in mammalian cells under glucose deprivation by phosphorylating ULK1 at Ser317 and Ser777 and the ATG6 homolog BECLIN-1 at Thr388 (Kim *et al.*, 2011; D. Zhang *et al.*, 2016). In plants, ATG1a is phosphorylated upon nutrient deficiency (Suttangkakul *et al.*, 2011) and this phosphorylation event is enhanced in KIN10 overexpressing lines (Chen *et al*., 2017), suggesting that KIN10 most likely acts upstream of autophagy to directly or indirectly phosphorylate ATG1. Under prolonged carbon starvation, KIN10 directly phosphorylates ATG6 to promote autophagy (Huang *et al*., 2019), consistent with findings in mammalian cells (D. Zhang *et al.*, 2016), and this regulation is independent of TORC. Upon endoplasmic reticulum stress and oxidative stress, SnRK1 is required for autophagy but TORC repression is not (Pu *et al.*, 2017a; Soto-Burgos and Bassham, 2017). Furthermore, the activation of autophagy by phosphate deprivation, which is associated with endoplasmic reticulum stress, is mediated by a TORC-independent pathway (Naumann *et al.*, 2019). Together, these results indicate that SnRK1 positively regulates autophagy by phosphorylating RAPTOR1B, which blocks the TORC signaling pathway, and/or through ATG1a and ATG6 phosphorylation, which results in autophagy activation. The phosphorylation sites targeted by KIN10 are, however, yet to be identified in these substrates.

#### Other kinases linked to autophagy regulation

SnRK2s are plant-specific serine or threonine kinases that play a critical role in abscisic acid (ABA) signaling pathways mediated by PYRABACTIN RESISTANCE 1/PYR1-LIKE (PYL) ABA receptors and protein phosphatase 2Cs (PP2Cs) (Hasan *et al.*, 2022). The Arabidopsis SnRK2 family contains 10 members and is divided into three subclasses, I, II, and III, but only subclass II (SnRK2.7 and SnRK2.8) and III (SnRK2.2, SnRK2.3, and SnRK2.6) are classified as ABA-responsive SnRK2s (Kamiyama *et al.*, 2021). When ABA accumulates under stress such as drought, binding of ABA to its receptors inhibits PP2C family proteins, which include ABI1, ABI2, HAB1, HAB2, PP2CA, and AHG1, and thus activates SnRK2 to phosphorylate downstream effectors (Umezawa *et al.*, 2013; Wang *et al*., 2013). Under stress, SnRK2s can phosphorylate RAPTOR and inactivate TORC, which would be predicted to allow autophagy activation (Wang *et al*., 2018). Under non-stressed conditions, TORC kinase phosphorylates PYL ABA receptors, preventing the PYL receptors from binding to ABA and PP2Cs. This allows PP2C to directly dephosphorylate ABA-activated SnRK2 and inactivate its kinase activity (Wang *et al*., 2018). The PP2C–SnRK2 interaction also causes the sequestration of SnRK1, resulting in the formation of a trimeric SnRK1 suppressor complex. Together, these events promote the TORC signaling pathway, which increases plant growth and suppresses stress responses (Wang *et al*., 2018; Belda-Palazón *et al.*, 2020). Under stress conditions, ABA accumulates and binds the PYL receptors, causing them to sequester PP2Cs from the PP2C–SnRK2–SnRK1 suppressor complex, thus dissociating the complex. SnRK1 and SnRK2 are then free to inhibit TORC in a phosphorylation-dependent manner to prevent growth and promote stress responses (Wang *et al*., 2018; Belda-Palazón *et al.*, 2020). Autophagy can be activated during stress even when sugars are abundant (Janse van Rensburg *et al.*, 2019), suggesting that inhibition of TORC by other kinases could induce autophagy. Whether or not the key modulators in ABA signaling, including SnRK2 (and probably PP2C), have emerged to induce autophagy under sugar excess or in response to stress-induced ABA accumulation is of interest for future study (Signorelli *et al.*, 2019).

BR is a phytohormone that plays important roles in plant growth, development, and stress response (Manghwar *et al.*, 2022). Increasing evidence links BR signaling and autophagy. The BRASSINOSTEROID INSENSITIVE 1 (BRI1)-EMS-SUPPRESSOR 1 (BES1) is a positive transcriptional regulator of BR response genes that promotes growth in Arabidopsis (Clouse, 2011). Upon carbon starvation or drought stress, the BIN2 kinase, which functions as a negative regulator of BR signaling, phosphorylates DOMINANT SUPPRESSOR OF KAR 2 (DSK2), a selective autophagy receptor for BES1. This promotes the interaction of DSK2 with ATG8 and induces BES1 degradation via selective autophagy (Nolan *et al.*, 2017). BIN2 also phosphorylates BRASSINAZOLE-RESISTANT 1 (BZR1), a paralog of BES1, to reduce its abundance and suppress BR signaling (He *et al.*, 2002). TORC enhances BZR1 protein accumulation to promote growth in Arabidopsis. However, TORC inactivation under carbon starvation causes autophagy-mediated degradation of BZR1 to balance growth and carbon availability (Z. Zhang *et al.*, 2016). TORC promotes BR-induced hypocotyl growth, and BR signaling suppresses autophagy, along with enhanced phosphorylation of ATG13a. BIN2-mediated phosphorylation of RAPTOR1B at Ser916 significantly activates autophagy and inhibits the phosphorylation of ATG13a by TORC (Liao *et al.*, 2023). In addition, quantitative phosphoproteomics and transcriptomic analysis showed that *bin2* and *raptor1b* mutants affect a common set of genes involved in growth and stress responses (Montes *et al.*, 2022). Collectively, these results strongly suggest that BIN2-mediated phosphorylation can positively influence autophagy activation in Arabidopsis in two main ways: (i) by destabilizing BES1/BZR1 leading to reduced BR signaling and/or (ii) by inhibiting TORC activity, which can decrease BZR1 abundance, reduce BR signaling, and block ATG13a phosphorylation leading to autophagy activation.

BAK1 is a receptor-like kinase that plays key roles in regulating BR signaling, programmed cell death and immune responses. BAK1 is also a dual-specificity kinase and can therefore phosphorylate both tyrosine and serine/threonine-containing substrates (He *et al.*, 2007; Kemmerling *et al.*, 2007; Wang *et al*., 2008; Oh *et al.*, 2009; Roux *et al.*, 2011; Shang *et al.*, 2021). The sustained expression of ATG18a is required for autophagy induction (Xiong *et al.*, 2005) and plant resistance to the necrotrophic fungus *Botrytis cinerea* (Lai *et al.*, 2011). BAK1 phosphorylates ATG18a at four sites, Thr241, Ser328, Ser361, and Thr387, which in turn suppresses autophagy and resistance to *B. cinerea* infection (Zhang *et al.*, 2021). A phosphomimic mutant of ATG18a at five sites, including Ser344 and all four BAK1 phosphosites, suppresses autophagosome formation and results in reduced autophagic activity. In contrast, overexpression of phosphonull ATG18a enhances autophagic activity and complements the decreased resistance of *atg18a* mutants to *B. cinerea* (Zhang *et al.*, 2021). The phosphorylation of the fifth phosphosite, Ser344, appears to be independent of BAK1 (Zhang *et al.*, 2021), and whether or not another kinase phosphorylates this site remains to be investigated.

### Protein phosphatases regulate autophagy

The PROTEIN PHOSPHATASE 2A (PP2A) is a serine/threonine protein phosphatase universally found in eukaryotes. The active form of PP2A is a heterotrimeric complex consisting of a scaffold (A) subunit, a regulatory (B) subunit, and a catalytic (C) subunit. All three subunits can have multiple isoforms with the catalytic subunit typically having the fewest (Máthé *et al.*, 2021). In yeast, PP2A, in complex with either CELL DIVISION CYCLE 55 (CDC55) or ROX THREE SUPPRESSOR 1 (RTS1) regulatory subunit, dephosphorylates ATG13 upon nutrient depletion or TORC inactivation to induce autophagy (Yeasmin *et al.*, 2016). To inhibit autophagy, TORC1 phosphorylates another regulatory subunit called TYPE 2A PHOSPHATASE-ASSOCIATED PROTEIN 42 (TAP42), which competitively binds to PP2A to repress PP2A–CDC55 and PP2A-RTS1 complex formation and possibly change PP2A substrate specificity. TAP42 is dephosphorylated upon TORC1 inactivation allowing the formation of the PP2A–CDC55 and PP2A–RTS1 complexes (Jiang and Broach, 1999; Yeasmin *et al.*, 2016). In plants, TAP46 (Arabidopsis homolog of TAP42) is phosphorylated by TORC *in vitro* and functions as a downstream positive effector of the TORC signaling pathway (Ahn *et al.*, 2011, 2015). Decreasing TAP46 expression mimics typical TORC inactivation phenotypes including a decrease in global translation and an increase in autophagic activity and nitrogen remobilization (Ahn *et al.*, 2011). Together, these findings demonstrate that TAP46 is phosphorylated by TORC to inhibit autophagy and promote TORC-related downstream effects in plants. However, unlike in yeast, the connections between TORC, TAP46, PP2A, ATG13, and autophagy are yet to be established in plants. The TYPE ONE PROTEIN PHOSPHATASE (TOPP) is another serine/threonine phosphatase involved in autophagy regulation. TOPP acts as an upstream component of autophagy and directly dephosphorylates ATG13a in Arabidopsis (Wang *et al*., 2022). Eighteen phosphorylation sites were identified in ATG13a. Phosphonull mutation of all of these sites increases tolerance to carbon starvation and facilitates ATG1/ATG13 complex formation. ATG13a dephosphorylation by TOPP increases ATG1a phosphorylation (Wang *et al*., 2022), suggesting that TOPP regulates autophagy by controlling the phosphorylation status of ATG1 complex components, and thereby its complex formation and activity.

### Persulfidation

Over the past decade, emerging evidence has suggested that hydrogen sulfide (H_2_S) is an important signaling molecule that regulates various molecular and developmental processes in plants (Dooley *et al.*, 2013; Aroca *et al.*, 2018; Wang *et al*., 2021; H. Li *et al*., 2022a). One of the main signaling mechanisms by which H_2_S regulates protein function is through a cysteine-dependent post-translational modification called persulfidation (also known as sulfhydration), in which cysteine thiols (R-SH) of a protein are modified to persulfide (R-SSH) (Mustafa *et al.*, 2009; Aroca *et al.*, 2015). More than 5000 proteins can undergo persulfidation in plants including those involved in the autophagy process (Aroca *et al.*, 2017; Jurado-Flores *et al.*, 2021), indicating a role for persulfidation in the regulation of plant autophagy.

In Arabidopsis, a role for sulfide as a signaling molecule that negatively regulates autophagy has been established over the past decade (Álvarez *et al.*, 2012; Laureano-Marín *et al.*, 2016, 2020; Aroca *et al.*, 2017, 2021). The enzyme L-CYSTEINE DESULFHYDRASE1 (DES1) is involved in sulfide metabolism in the cytosol and catalyses the production of H_2_S from cysteine to maintain H_2_S homeostasis in plant cells (Alvarez *et al.*, 2010, 2012; Jin *et al.*, 2011). *des1* mutants exhibit reduced endogenous sulfide levels. This is accompanied by increased *ATG8b* and *ATG12a* transcripts along with increased ATG8 protein abundance and lipidation (Álvarez *et al.*, 2012). Exogenous application of sulfide reverses these effects in the *des1* mutant, suggesting that H_2_S negatively regulates autophagy by suppressing *ATG* gene expression and decreasing ATG8 lipidation (Álvarez *et al.*, 2012). Moreover, *DES1* expression is significantly down-regulated upon nitrogen starvation as a means to promote autophagy (Laureano-Marín *et al.*, 2016). Since *des1* mutants possess low endogenous H_2_S levels (Álvarez *et al.*, 2012), persulfidation of ATG proteins such as ATG2, 3, 4, 5, 7, 11, and 13 (Jurado-Flores *et al.*, 2021) in the cytosol is likely reduced in the mutant. Additional studies confirming this hypothesis will further indicate the extent to which H_2_S suppresses autophagy via the persulfidation of several ATG proteins in a DES1-dependent manner.

The reversible modification of ATG4a at Cys170 and ATG18a at Cys103 by persulfidation inhibits autophagosome formation and suppresses autophagy (Laureano-Marín *et al.*, 2020; Aroca *et al.*, 2021). ATG4 is a protease that cleaves the C-terminus of the inactive ATG8 precursor to expose a glycine, prior to ATG8 conjugation to phosphatidylethanolamine (Yoshimoto *et al.*, 2004). ATG4a is highly persulfidated under normal growth conditions, inhibiting its proteolytic activity. In response to ABA, nitrogen starvation, or osmotic stress, the persulfidation of ATG4a at Cys170 is substantially reduced resulting in an increase in its proteolytic activity and ATG8 maturation (Laureano-Marín *et al.*, 2020). Under endoplasmic reticulum stress, persulfidation of ATG18a at Cys103 increases its membrane- and lipid-binding affinity, delaying its release from the growing phagophore, and significantly suppressing autophagosome formation. In contrast, abolishing persulfidation at Cys103 decreases the membrane- and lipid-binding affinity of ATG18a, decreasing autophagosome size but increasing autophagosome number. Differential persulfidation of ATG18a could therefore serve as a means to properly regulate autophagosome biogenesis to facilitate an appropriate level of autophagic response to endoplasmic reticulum stress (Aroca *et al.*, 2021).

Recently, a report showed H_2_S as a positive regulator of autophagy by up-regulating the expression of *ATG* genes upon submergence, thus alleviating cell death (Xuan *et al.*, 2022). The role of H_2_S as a positive or negative regulator might depend on the particular stress encountered, and possibly involve other regulators in the autophagy signaling pathway. Indeed, in mammals, sulfide has been shown to activate or suppress autophagy, depending on the context (Wu *et al*., 2018). Therefore, it is not surprising that sulfide could also exhibit opposing effects on plant autophagy.

### Ubiquitination

Ubiquitination is a post-translational modification in which a 76 amino acid ubiquitin polypeptide, around 8.6 kDa, is covalently bonded to a lysine residue of the target protein. In yeast and mammals, ubiquitination of key components of autophagy, for instance ATG1 (ULK1 in mammals) (Nazio *et al.*, 2013) and ATG6 (BECLIN 1 in mammals) (Shi and Kehrl, 2010; Xia *et al.*, 2013), controls multiple steps in autophagy. Several studies in Arabidopsis show that stability of ATG1/ATG13 complex subunits and ATG6 are regulated by both autophagy and proteasomal degradation (Suttangkakul *et al.*, 2011; Qi *et al.*, 2017, 2020).

In mammals, the E3 ligase TUMOR NECROSIS FACTOR RECEPTOR ASSOCIATED FACTOR 6 (TRAF6) functions as a signaling adaptor to mediate K63-linked ubiquitination of ULK1. This stabilizes ULK1 by activating its self-association and kinase activity, thus activating autophagy (Nazio *et al.*, 2013). In Arabidopsis, TRAF1a and TRAF1b recruit the RING-finger E3 ligases SEVEN IN ABSENTIA OF *ARABIDOPSIS THALIANA* 1 (SINAT1) and SINAT2 under normal growth conditions to facilitate the ubiquitination and degradation of ATG6 and ATG13 (Qi *et al.*, 2017, 2020), thereby maintaining low autophagic activity. Conversely, under nutrient deprivation, SINAT6 disrupts the interaction between TRAF1a/1b and SINAT1/2 by competitively associating with ATG6 and ATG13. This stabilizes ATG6 and the ATG1/ATG13 complex resulting in autophagy activation (Qi *et al.*, 2017, 2020). Together, these findings indicate that plant TRAF proteins dynamically mediate autophagy by interacting with different SINAT proteins to modulate ATG6 and ATG13 protein stability under normal or stress conditions. Moreover, ATG1a-mediated phosphorylation of TRAF1a under nutrient starvation promotes TRAF1a stability, suggesting a feedback regulatory mechanism between the ATG1/ATG13 kinase complex and TRAF1 proteins (Qi *et al.*, 2020).

The 14-3-3 proteins are a family of regulatory proteins that specifically recognize, bind, and control the activity of a wide array of phosphorylated target proteins in plants (Camoni *et al.*, 2018; Zhao *et al.*, 2021; Huang *et al.*, 2022). In autophagy, the 14-3-3λ and 14-3-3κ proteins function as adaptors to mediate SINAT1/2-dependent ubiquitination and degradation of phosphorylated ATG13a under nutrient-sufficient conditions (Qi *et al.*, 2022). This suggests that the formation of a TRAF1a/1b–SINAT1/2-(14-3-3λ/κ)–ATG13 complex is essential for ATG13 degradation and autophagy inhibition while the formation of a trimeric TRAF1–SINAT6–ATG13 complex is required to stabilize the ATG1/ATG13 kinase complex for autophagy induction. An ATG13a phosphonull mutant, harboring alanine substitutions in 18 putative phosphorylation sites, displays decreased interaction with 14-3-3λ while its phosphomimic variant exhibits increased interaction with 14-3-3λ (Qi *et al.*, 2022). Interestingly, some of the mutated sites such as Ser248, Ser397, Ser404, Ser406, and Ser407 are associated with the TORC kinase (Van Leene *et al.*, 2019), suggesting that TORC-mediated phosphorylation may negatively regulate ATG13 in a 14-3-3-dependent manner to suppress autophagy. In Arabidopsis, ATG6 accumulates under short-term carbon starvation, but its levels are reduced via the 26S proteasome under long-term carbon starvation (Qi *et al.*, 2017). SnRK1 phosphorylates ATG6 during prolonged carbon starvation to promote autophagy (Huang *et al*., 2019). It is, however, not clear whether SnRK1-mediated phosphorylation of ATG6 promotes its ubiquitination and degradation under long-term carbon starvation and whether the 14-3-3 proteins have a role to play in this process.

SH3 DOMAIN-CONTAINING PROTEIN 2 (SH3P2) is a BIN-AMPHIPHYSIN-RVS (BAR) domain-containing protein that localizes to the phagophore assembly site to engage in membrane remodeling events during autophagosome biogenesis (Zhuang *et al.*, 2013). SH3P2 interacts with the PI3K complex and ATG8 to promote autophagosome expansion and maturation and is required for the delivery of autophagosomes to the vacuole (Zhuang *et al.*, 2013). In addition, SH3P2 interacts with the ubiquitin-binding protein FYVE DOMAIN PROTEIN REQUIRED FOR ENDOSOMAL SORTING 1 (FREE1) to promote autophagosome–vacuole fusion and autophagic degradation (Gao *et al.*, 2014, 2015). Interestingly, the bacterial effector E3 ligase XopL ubiquitinates SH3P2 and targets it for proteasomal degradation to suppress autophagy and facilitate *Xanthomonas campestris* pv*. vesicatoria* (*Xcv*) infection of plant hosts (Leong *et al.*, 2022). These results hint that the ubiquitination of SH3P2 by a plant E3 ligase and its subsequent proteasomal degradation could serve as a means to negatively regulate SH3P2 stability and autophagy induction under non-stressed conditions.

### Other potential post-translational modifications in autophagy regulation

Other possible post-translational modifications may function in autophagy but have yet to be well studied in plants. Nitric oxide acts as a signaling molecule through the post-translational modification *S*-nitrosylation (Astier *et al.*, 2011), and regulates autophagy in animals (Montagna *et al.*, 2016; Tegeder, 2019; Ma *et al.*, 2020; Liu *et al*., 2022). While regulation of autophagy by nitric oxide in plants is not clear, autophagic substrate recognition has been shown to depend on *S*-nitrosylation. *S*-nitrosylation of Arabidopsis GSNO REDUCTASE 1 (GSNOR1) induces a conformational change in its ATG8-interacting motif (AIM) motif. This allows GSNOR1 to interact with ATG8 leading to its degradation via selective autophagy in response to hypoxia (Zhan *et al.*, 2018). A role for protein acetylation in regulating autophagy is well-established in yeast and mammals (Lee *et al.*, 2008; McEwan and Dikic, 2011; Yi *et al.*, 2012; Huang *et al*., 2015; Su *et al.*, 2017). For example, in mammals, p300-mediated acetylation and SIRTUIN1 (SIRT1)-mediated deacetylation of MICROTUBULE-ASSOCIATED PROTEIN 1A/1B-LIGHT CHAIN 3 (LC3), the mammalian homolog of ATG8, regulates its nucleocytoplasmic transport and activity to either inhibit or stimulate autophagy, respectively (Lee *et al.*, 2008; Huang & Liu, 2015; Huang *et al*., 2015). In the silkworm, *Bombyx mori*, p300-mediated acetylation of members of the ATG8 ubiquitin-like conjugation system, BmATG3, BmATG4, BmATG7, and BmATG8, inhibits autophagy while HISTONE DEACETYLASE 1 (HDAC1)-mediated deacetylation of these ATG proteins yields the opposite effect (Wu *et al.*, 2021a). Interestingly, TORC1 directly phosphorylates and activates p300 to inhibit autophagy under normal conditions in mammals (Wan *et al.*, 2017) while cholesterol-mediated inactivation of TORC1 promotes the dephosphorylation of HDAC1 to stimulate autophagy in the silkworm (Wu *et al.*, 2021b), indicating a key role for TORC-dependent phosphorylation in acetylation-mediated regulation of autophagy. Currently, studies on the role of acetylation in autophagy regulation in plants are scarce, suggesting a knowledge gap that needs to be addressed. In particular, it will be interesting to know if regulation of ATG proteins via acetylation and deacetylation mechanisms, as observed in other species, is conserved in plants and whether plant TORC plays a role in mediating these events.

## Transcriptional regulation of plant autophagy

**Fig. 3.**
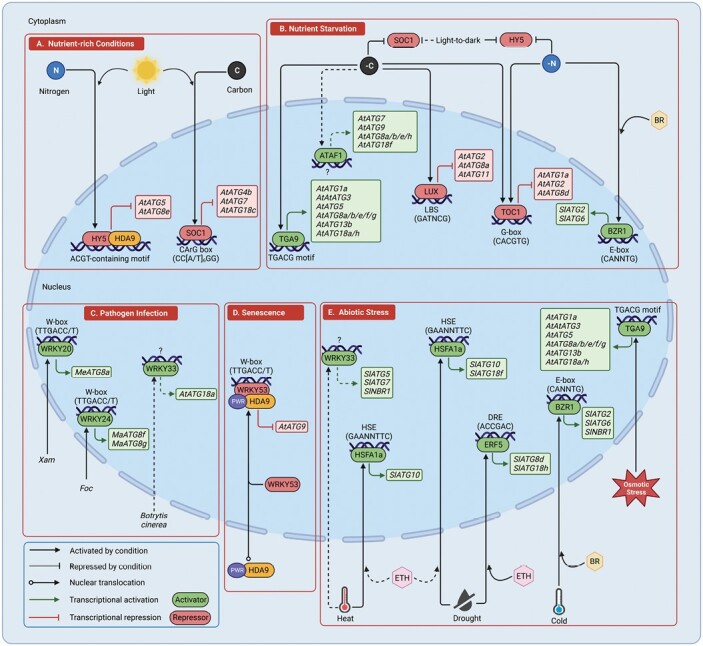
Model of the role of transcriptional regulators of plant autophagy. (A) Under nutrient-rich conditions, HY5 and SOC1 translocate to the nucleus where they repress *ATG* expression to maintain autophagy at a low basal level. (B) Under nutrient starvation, HY5 and SOC1 protein abundance are reduced, which negatively regulates their transcriptional activities. In Arabidopsis, positive regulators such as TGA9 and ATAF1 up-regulate several *ATG* genes to promote autophagy and enhance starvation tolerance. Negative regulators such as LUX and TOC1 down-regulate *ATG* expression to fine-tune the level of autophagy and prevent autophagy-induced cell death. In tomato plants, BR induces the translocation of BZR1 into the nucleus where it activates *ATG* expression in response to starvation. (C) WRKY transcription factors positively regulate *ATG* expression to facilitate autophagy-mediated resistance to pathogen infection in various plant species. (D) To promote leaf senescence, WRKY53, in complex with PWR and HDA9, represses *ATG9* expression. (E) Several transcription factors positively regulate *ATG* expression in response to different abiotic stresses such as heat (WRKY33, HSFA1a), drought (HSFA1a, ERF5), cold (BZR1), and osmotic stress (TGA9). Solid lines indicate direct transcriptional regulation with experimental evidence. Dashed lines indicate direct transcriptional regulation requiring experimental validation. BR, brassinosteroid; ETH, ethylene; *Foc*, *Fusarium oxysporum* f. sp. *cubense*; *Xam*, *Xanthomonas axonopodis* pv. *manihotis*.

While the immediate and rapid activation of autophagy by stress is dependent on post-translational events, under non-optimal conditions plants increase the expression of *ATG* genes to sustain autophagic activity and improve stress tolerance (Xiong *et al.*, 2005; Rose *et al.*, 2006; Xia *et al.*, 2011; Zhou *et al.*, 2013; Wang *et al*., 2015). Transcriptional control is therefore required to allow the proper expression of *ATG* genes in response to stress and developmental changes. Some transcriptional regulators of *ATG* genes have been identified and characterized (Yan *et al.*, 2017; Wang *et al*., 2020; Yang *et al.*, 2020; Chen *et al.*, 2022), providing a glimpse of the transcriptional mechanisms underlying autophagy gene regulation in plants (Fig. 3; Table 1).

**Table 1.** Transcription factors and their target genes in autophagy regulation

Transcription factor	ATG target	Putative roles in autophagy	References
Positive regulators			
ATAF1^*a*^	*AtATG7*	ATG8 lipidation	Garapati *et al.* (2015)
	*AtATG9*	Lipid source	
	*AtATG8a/b/e/h*	Autophagosome maturation, cargo recognition	
	*AtATG18f*	PI3P effector	
BZR1	*SlATG2*	Membrane expansion	Wang *et al*. (2019), Chi *et al.* (2020)
	*SlATG6*	Vesicle nucleation	
	*SINBR1*	Autophagy receptor	
ERF5	*SlATG8d*	Autophagosome maturation, cargo recognition	Zhu *et al.* (2018)
	*SlATG18h*	PI3P effector	
HSFA1a	*SlATG10*	ATG12 conjugation	Wang *et al*. (2015)
	*SlATG18f*	PI3P effector	
TGA9	*AtATG1a*	Phagophore induction	Wang *et al*. (2020)
	*AtAtATG3*	ATG8 lipidation	
	*AtATG5*	ATG8 lipidation	
	*AtATG8a/b/e/f/g*	Autophagosome maturation, cargo recognition	
	*AtATG13b*	Phagophore induction	
	*AtATG18a/h*	PI3P effector	
WRKY20	*MeATG8a*	Autophagosome maturation, cargo recognition	Yan *et al.* (2017)
WRKY24	*MaATG8f/g*	Autophagosome maturation, cargo recognition	Liu *et al.* (2019)
WRKY33^*a*^	*AtATG18*	PI3P effector	Lai *et al.* (2011), Zhou *et al.* (2014)
	*SIATG5*	ATG8 lipidation	
	*SIATG7*	ATG8 lipidation	
	*SINBR1*	Autophagy receptor	
Negative regulators			
HY5	*AtATG5*	ATG8 lipidation	Yang *et al.* (2020)
	*AtATG8e*	Autophagosome maturation, cargo recognition	
LUX	*AtATG2*	Membrane expansion	Yang *et al.* (2022)
	*AtATG8a*	Autophagosome maturation, cargo recognition	
	*AtATG11*	Phagophore induction	
SOC1	*AtATG4b*	ATG8 maturation	X. Li *et al*. (2022)
	*AtATG7*	ATG8 lipidation	
	*AtATG18c*	PI3P effector	
TOC1	*AtATG1a*	Phagophore induction	Chen *et al.* (2022)
	*AtATG2*	Membrane expansion	
	*AtATG8d*	Autophagosome maturation, cargo recognition	
WRKY53	*AtATG9*	Lipid source	Chen *et al.* (2016)

^*a*^ Not experimentally confirmed to directly regulate *ATG* genes.

### WRKY transcription factors

The WRKY proteins form one of the largest transcription factor families in plants. Members of this group are well-studied and play important roles in regulating stress response pathways (Chen *et al.*, 2019), including the autophagy process. In Arabidopsis, WRKY53, in complex with HISTONE DEACETYLASE 9 (HDA9) and POWERDRESS (PWR), binds to the W-box motif in the *ATG9* promoter and suppresses its expression in an H3K27 deacetylation-dependent manner (Chen *et al.*, 2016). This promotes leaf senescence in Arabidopsis, consistent with the early leaf senescence phenotype of *atg9* mutants (Hanaoka *et al.*, 2002; Chen *et al.*, 2016). Infection by the necrotrophic fungus *Botrytis cinerea* induces autophagy in both infected and surrounding uninfected cells of Arabidopsis plants, along with increased expression of *WRKY33* and *ATG* genes (Zheng *et al.*, 2006; Lai *et al.*, 2011). Loss of WRKY33 enhances susceptibility to *Botrytis* infection while WRKY33 overexpression has the opposite effect (Zheng *et al.*, 2006). To restrict the spread of the infection, WRKY33 is required for autophagy in cells that surround the infected area (Lai *et al.*, 2011). One way in which WRKY33 may achieve this is by promoting the sustained expression of *ATG18a* (Lai *et al.*, 2011), a key protein in autophagosome formation (Xiong *et al.*, 2005). In addition, WRKY33 physically interacts with ATG18a in the nucleus (Lai *et al.*, 2011), possibly regulating WRKY33 transcriptional activity during pathogen infection. However, the significance of this interaction, and whether WRKY33 directly regulates *ATG18a* expression, is not clearly established. In tomato (*Solanum lycopersicum*), silencing WRKY33 decreases the expression of *ATG5*, *ATG7*, and *NEIGHBOR OF BRCA1 GENE* (*NBR1*). It also compromises autophagosome formation, leading to increased accumulation of insoluble protein aggregates and decreased heat stress tolerance in tomato plants (Zhou *et al.*, 2014). Since NBR1 facilitates selective autophagy (Zhou *et al.*, 2013), WRK33 may promote selective degradation and recycling of heat-induced protein aggregates by enhancing NBR1 expression.

In cassava (*Manihot esculenta*), bacterial blight caused by *Xanthomonas axonopodis* pv. *manihotis* (*Xam*) infection induces expression of MeWRKY20, which then translocates to the nucleus to directly activate the expression of *ATG8a* (Yan *et al.*, 2017). This enhances autophagic activity and increases callose deposition in the cell wall to reinforce a physical barrier, restricting the spread of the infection (Yan *et al.*, 2017). Moreover, MeWRKY20 physically interacts with MeATG8a/8f/8h (Yan *et al.*, 2017), suggesting a possible feedback mechanism where the protein abundance of pathogen-induced MeWRKY20 is controlled via the autophagy pathway. Similarly, in banana (*Musa acuminata*), *Fusarium oxysporum* f. sp. *cubense* (*Foc*) infection induces expression of WRKY24, which directly activates the expression of *MaATG8f* and *MaATG8g* by binding to the W-box in their promoters (Liu *et al*., 2019). Overexpressing *MaATG8f* and *MaATG8g* increases autophagosome formation in *Foc*-inoculated Arabidopsis plants. However, overexpressing *MaWRKY24*, *MaATG8f* and *MaATG8g* in Arabidopsis increases disease susceptibility to *Foc* (Liu *et al*., 2019), and transient expression of *MaATG8* in tobacco (*Nicotiana benthamiana*) leads to a hypersensitive-like cell death phenotype (Wei *et al.*, 2017). This suggests that MaWRKY24-mediated *MaATG8f* and *MaATG8g* expression may contribute to disease susceptibility to *Foc* by inducing autophagy-dependent cell death.

### ATAF1 and TGA9

Transcription factors also play a key role in starvation-induced autophagic responses in plants. Overexpression of the NAM, ATAF and CUC (NAC)-domain protein ARABIDOPSIS TRANSCRIPTION ACTIVATION FACTOR1 (ATAF1) reprograms the Arabidopsis transcriptome to mimic carbon starvation, with elevated expression of genes involved in starch and amino acid catabolism as well as autophagy (Garapati *et al.*, 2015). ATAF1-induced *ATG* genes include *ATG7*, *ATG8a/8b/8e/8h*, *ATG9*, and *ATG18a*. In contrast, decreased ATAF1 levels result in the down-regulation of these *ATG* genes (Garapati *et al.*, 2015). Interestingly, ATAF1 interacts with Arabidopsis SnRK1 catalytic subunits KIN10 and KIN11 (Kleinow *et al.*, 2009), suggesting that ATAF1 is a potential phosphorylation target for SnRK1-mediated regulation. These findings together suggest that ATAF1 may play a role in the regulation of starvation-induced autophagy, but further studies are required to confirm this. Recently, the basic leucine zipper (bZIP) protein TGACG (TGA) MOTIF-BINDING PROTEIN 9 (TGA9) was identified as a positive modulator of autophagy-dependent carbon starvation responses in Arabidopsis (Wang *et al*., 2020). TGA9 promotes autophagy-dependent tolerance to carbon starvation by directly binding to the TGACG motif-containing promoters of several *ATG* genes such as *ATG1a*, *ATG5*, *ATG8a/8f/8g*, and *ATG18h*, and up-regulating their expression (Wang *et al*., 2020). TGA9 also enhances the expression of several *ATG* genes to stimulate autophagy upon osmotic stress (Wang *et al*., 2020), but its contribution to osmotic stress tolerance is yet to be established.

### HY5 and SOC1

The ELONGATED HYPOCOTYL 5 (HY5) protein is a light-responsive bZIP transcription factor that promotes photomorphogenesis (Wei *et al.*, 1994) and functions as a negative regulator of autophagy (Yang *et al.*, 2020). HY5 inhibits autophagy under optimal growth conditions by down-regulating *ATG5* and *ATG8e*. HY5 controls *ATG* promoter activity by direct recruitment of HDA9 which, in turn, decreases H3K9/K27 acetylation levels to silence gene expression (Yang *et al.*, 2020). In response to light-to-dark conversion or nitrogen deficiency, HY5 is degraded via the 26S proteasome resulting in HDA9-promoter dissociation. This elevates H3K9/K27 acetylation levels and up-regulates *ATG* expression to activate autophagy and enhance stress tolerance (Yang *et al.*, 2020). Another negative regulator of starvation responses in plants is the MADS-box protein SUPPRESSOR OF OVEREXPRESSION OF CONSTANS 1 (SOC1), a known modulator of flowering time (Onouchi *et al.*, 2000; Liu *et al*., 2007). SOC1 suppresses autophagy by down-regulating *ATG4b*, *ATG7*, and *ATG18c*, which decreases tolerance of carbon starvation. SOC1 expression is suppressed under carbon starvation as a means to promote autophagy-dependent plant survival (X. Li *et al*., 2022). SOC1 also down-regulates abiotic stress responses during the floral transition process. Histone H4 acetylation of SOC1 chromatin by chromatin remodeling factor MORF-RELATED GENE (MRG) activates SOC1 expression leading to the up-regulation of floral regulator *LEAFY* (*LFY*) and the down-regulation of diverse stress-responsive genes. This results in decreased abiotic stress tolerance in Arabidopsis (Barrero-Gil *et al.*, 2021). Based on these findings, MRG may modulate SOC1-dependent regulation of autophagy and *ATG* gene expression. Interestingly, the MRG–SOC1 module bears some similarities to the HDA9–HY5 module. The transcription factor units of both modules, i.e. SOC1 and HY5, down-regulate *ATG* expression to inhibit autophagy, and their protein levels are substantially reduced in response to starvation (Yang *et al.*, 2020; X. Li *et al*., 2022). In contrast to the MRG–SOC1 module, the HDA9–HY5 module negatively regulates flowering time by repressing *PHYTOCHROME INTERACTING FACTOR 4* (*PIF4*) and *CONSTANS-LIKE 5* (*COL5*) in a histone deacetylation-dependent manner, possibly to fine-tune the floral transition process (Chu *et al.*, 2022). Together, these findings suggest that an MRG/SOC1–HDA9/HY5 signaling axis may exist to transcriptionally coordinate autophagy with flowering time.

### LUX and TOC1

Circadian regulation plays essential roles in plant responses to different biotic and abiotic signals (Salter *et al.*, 2003; Fowler *et al.*, 2005; Wang *et al*., 2011; Lai *et al.*, 2012; Mizuno *et al.*, 2014; Box *et al.*, 2015) and helps to modulate plant growth and metabolism in response to changes in the environment (Buckley *et al.*, 2023). Autophagy is influenced by the circadian clock and is rhythmically activated under both constant light and light–dark conditions through the rhythmic expression of *ATG* genes (Chen *et al.*, 2022; Yang *et al.*, 2022). In turn, a functional autophagy pathway is required to maintain the stability of the endogenous circadian rhythm in plants (Chen *et al.*, 2022). Under light/dark conditions, autophagic activity is highest at night. To prevent overactivation of autophagy, which could result in cell death (Kang *et al*., 2007), the circadian clock transcription factor LUX ARRHYTHMO (LUX) represses *ATG2*, *ATG8a*, and *ATG11* genes, thereby promoting tolerance of dark-induced starvation (Yang *et al.*, 2022). Moreover, LUX-mediated repression of *ATG* genes is required to maintain a normal autophagy rhythm, fine-tune autophagic response to carbon deficiency, and enhance survival under carbon starvation (Yang *et al.*, 2022). Another circadian clock transcription factor, TIMING OF CAB EXPRESSION 1 (TOC1), moderates the extent of autophagy under nutritional stress by suppressing expression of *ATG1a*, *ATG2*, and *ATG8d* genes. Importantly, TOC1 activity contributes to starvation tolerance in Arabidopsis (Chen *et al.*, 2022). Interestingly, both LUX and TOC1 are evening-expressed genes that are co-regulated by the circadian clock with similar expression patterns under light–dark conditions (Hazen *et al.*, 2005). LUX and TOC1 may therefore work together to moderate autophagy levels at night and during nutritional deficiency to promote plant survival.

### BZR1

The brassinolide activated BZR1 transcription factor positively regulates BR signaling and autophagy in tomato plants. BZR1 is dephosphorylated and activated in response to cold treatment and nitrogen starvation in tomato, where it up-regulates the expression of *ATG2* and *ATG6* to promote autophagy (Wang *et al*., 2019; Chi *et al.*, 2020). BZR1-mediated autophagy decreases stress-induced accumulation of insoluble protein aggregates, thereby increasing the tolerance of tomato plants to cold stress and nitrogen starvation (Wang *et al*., 2019; Chi *et al.*, 2020). Moreover, BZR1 enhances the expression of NBR1 under cold stress to facilitate the selective autophagy of accumulated protein aggregates (Chi *et al.*, 2020). It is also worth noting that BR alone elevates autophagic activity in non-stressed tomato plants and causes the enrichment of BZR1 in the promoters of *ATG2*, *ATG6*, and *NBR1* (Wang *et al*., 2019; Chi *et al.*, 2020). This indicates an important function for BZR1-mediated BR signaling in plant autophagy. In Arabidopsis, BZR1 appears to play an antithetical role in autophagy compared with its tomato counterpart. For instance, TORC promotes BZR1 stability to stimulate growth and upon TORC inactivation or carbon starvation, BZR1 and its paralog BES1 are degraded to promote stress response and tolerance (Z. Zhang *et al.*, 2016; Nolan *et al.*, 2017). This is in contrast to its tomato homolog which accumulates in response to nitrogen starvation (Wang *et al*., 2019). The activation and role of BZR1 in autophagy regulation may therefore be dependent on the type of stress and plant species, and further studies are required to shed light on this.

### HSFA1a and ERF5

The HEAT SHOCK TRANSCRIPTION FACTOR A1a (HSFA1a) and the ETHYLENE RESPONSE FACTOR 5 (ERF5) are induced by drought stress to activate autophagy in tomato (Pan *et al.*, 2012; Wang *et al*., 2015; Zhu *et al.*, 2018). HSFA1a directly binds heat-shock elements (GAANNTTC) in *ATG10* and *ATG18f* promoters to activate their expression (Wang *et al*., 2015) while ERF5 activates *ATG8d* and *ATG18h* expression by binding to drought-responsive elements (ACCGAC) in their promoters (Zhu *et al.*, 2018). HSFA1a-mediated autophagy reduces the accumulation of insoluble ubiquitinated protein aggregates to enhance drought resistance while ERF5-mediated autophagy contributes to ethylene-mediated drought tolerance in tomato (Wang *et al*., 2015; Zhu *et al.*, 2018). In addition, HSFA1a promotes pollen thermotolerance in tomato by up-regulating *ATG10* expression to stimulate autophagy and reduce heat stress-induced aggregated proteins (Xie *et al.*, 2022). In rice, ethylene-precursor treatment significantly increases HSFA1a expression under heat stress (Wu and Yang, 2019), suggesting a possible role for HSFA1a in ethylene-mediated autophagy induction under both heat and drought stress conditions.

## Conclusions and perspectives

**Fig. 4.**
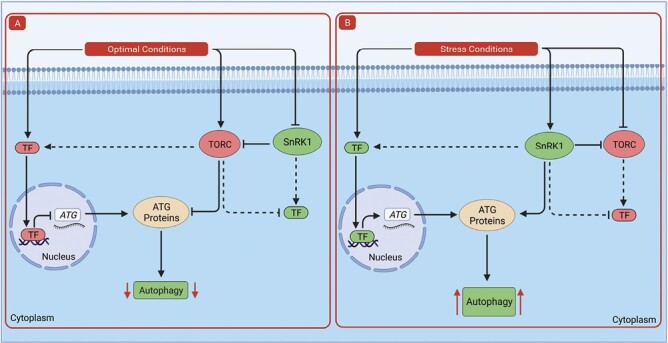
Proposed model of potential connections between transcriptional and post-translational regulation of autophagy. (A) Under optimal growth conditions, the activity of positive regulators of autophagy (e.g. ATAF1, SnRK1) is inhibited while negative regulators are activated. Negative upstream regulators, such as TORC, can control ATG protein abundance by suppressing the transcriptional activation of *ATG* expression through post-translational modifications of transcriptional regulators. In addition, these negative upstream regulators may inhibit the stability and function of ATG proteins in the cytosol and thereby keep autophagy at a low basal level. (B) In stress conditions, the activity of negative regulators (e.g. SOC1, HY5, TORC, SINAT1/2) is inhibited while positive regulators (e.g. TGA9, HSFA1a, SnRK1) are activated. Positive upstream regulators (e.g. SnRK1) can enhance ATG protein abundance by promoting the transcriptional activation of *ATG* expression. Upstream regulators can also promote the stability and function of ATG proteins in the cytosol leading to increased autophagic activity. Black lines with arrow heads indicate activation while black lines with bars indicate repression. Solid black lines indicate activation or repression with experimental evidence whereas dashed black lines indicate lack of experimental evidence. Red arrows pointing down indicate decreased autophagy while red arrows pointing up indicate increased autophagy.

Recent studies have established that plants possess a transcriptional and post-translational network of proteins that potentially work together in a context-dependent manner to fine-tune and regulate the plant autophagy process. Transcriptional regulation provides a means for plant cells to replenish the autophagy machinery, especially under prolonged starvation or stress periods, and also control the supply of autophagy proteins to maintain an appropriate level of stress response. Post-translational modifications control ATG protein abundance, stability, and function in response to upstream signals. Interestingly, studies have shown that changes in the levels of the two main upstream regulators of plant autophagy, i.e. SnRK1 and TORC, reprogram the plant transcriptome, with significant changes in genes involved in the autophagy process. This indicates a potential connection between transcriptional and post-translational regulation of autophagy (Fig. 4). Exploring this connection using large-scale approaches such as phosphoproteomics combined with genome-wide transcriptome analysis can provide a platform to further identify new regulators and more importantly to build a comprehensive network of how plant autophagy is regulated. In addition, a better understanding of such a regulatory network can enhance food security by accelerating future agronomic improvements, especially in tolerance of different types of stress conditions.

## Abbreviations:

ABA: abscisic acid
AMPK: AMP-ACTIVATED PROTEIN KINASE
ATAF1: ARABIDOPSIS TRANSCRIPTION ACTIVATION FACTOR 1
ATG: autophagy-related
BAK1: BRASSINOSTEROID INSENSITIVE 1-ASSOCIATED RECEPTOR KINASE 1
BES1: BRASSINOSTEROID INSENSITIVE 1 (BRI1)-EMS-SUPPRESSOR 1
BIN2: BRASSINOSTEROID-INSENSITIVE 2
BR: brassinosteroid
BZR1: BRASSINAZOLE-RESISTANT 1
CDC55: CELL DIVISION CONTROL PROTEIN 55
DES1: L-CYSTEINE DESULFHYDRASE
DSK2: DOMINANT SUPPRESSOR OF KAR 2
ERF5: ETHYLENE RESPONSE FACTOR 5
FREE1: FYVE DOMAIN PROTEIN REQUIRED FOR ENDOSOMAL SORTING 1
GSNOR1: GSNO REDUCTASE 1
HDA9: HISTONE DEACETYLASE 9
HSFA1A: HEAT SHOCK TRANSCRIPTION FACTOR A1A
HY5: ELONGATED HYPOCOTYL 5
KIN10: SNF1 KINASE HOMOLOG 10
LST8: LETHAL WITH SEC THIRTEEN 8
LUX: LUX ARRYTHMO
NBR1: NEIGHBOR OF BRCA1 GENE
PI3K: PHOSPHATIDYLINOSITOL 3-KINASE
PP2A: PROTEIN PHOSPHATASE 2A
PP2C: PROTEIN PHOSPHATASE 2C
RAPTOR: REGULATORY-ASSOCIATED PROTEIN OF TOR
RTS1: ROX THREE SUPPRESSOR 1
SH3P2: SH3 DOMAIN-CONTAINING PROTEIN 2
SINAT: SEVEN IN ABSENTIA OF *ARABIDOPSIS THALIANA* 1
SNRK: SNF-RELATED KINASE
SOC1: SUPPRESSOR OF OVEREXPRESSION OF CONSTANS 1
TAP42: TYPE 2A PHOSPHATASE-ASSOCIATED PROTEIN 42
TAP46: TYPE 2A PHOSPHATASE-ASSOCIATED PROTEIN 46
TGA9: TGACG MOTIF-BINDING PROTEIN 9
TOC1: TIMING OF CAB EXPRESSION 1
TOPP: TYPE ONE SERINE/THREONINE PROTEIN PHOSPHATASE
TORC: target of rapamycin complex
TRAF: TUMOR NECROSIS FACTOR RECEPTOR ASSOCIATED FACTOR
ULK1: UNC51-LIKE AUTOPHAGY ACTIVATING KINASE 1
